# Long-Term Disease Control with Triapine-Based Radiochemotherapy for Patients with Stage IB2–IIIB Cervical Cancer

**DOI:** 10.3389/fonc.2014.00184

**Published:** 2014-07-24

**Authors:** Charles A. Kunos, Tracy M. Sherertz

**Affiliations:** ^1^Department of Radiation Oncology, Summa Cancer Institute, Summa Health System, Akron, OH, USA; ^2^Department of Radiation Oncology, CASE Comprehensive Cancer Center, University Hospitals Case Medical Center, Case Western Reserve University School of Medicine, Cleveland, OH, USA

**Keywords:** triapine, cervical cancer, ribonucleotide reductase, radiation, cisplatin

## Abstract

**Background:** National Cancer Institute phase I #7336 and phase II #8327 clinical trials explored the safety and efficacy of triapine (NSC #663249) added to cisplatin radiochemotherapy in untreated patients with advanced-stage cervical cancer. Triapine inhibits ribonucleotide reductase, the rate-limiting enzyme responsible for DNA-building deoxyribonucleotides, and thereby, enhances radiochemosensitivity by prolonging DNA repair time. Here, we report 3-year efficacy endpoints of pelvic locoregional relapse rate, disease-free, and overall survivals.

**Methods:** Eligible patients with bulky IB–IIIB cervical cancer underwent three-times weekly triapine (25 or 50 mg/m^2^), once-weekly cisplatin (40 mg/m^2^), and conventional daily pelvic radiation followed by brachytherapy. A cumulative incidence method estimated pelvic locoregional relapse rates. Disease-free survival was measured from radiochemotherapy start date to the date of first relapse or cancer-related death. Overall survival was measured from radiochemotherapy start date to the date of any-cause death. The Kaplan–Meier method estimated survivals.

**Findings:** Between 2006 and 2011, 24 untreated patients with cervical cancer met criteria for reporting in this study. A median 3.4 years of follow-up time (range, 0.3–7.6 years) has been observed. All had squamous cancers and the majority had either node-positive stage IB–IIA (33%) or stage IIIB (42%) disease. The 3-year pelvic locoregional relapse rate, disease-free survival, and overall survival were 4% [95% confidence interval (CI), 0–20%], 80% (95% CI: 71–89%), and 82% (95% CI: 74–90%), respectively.

**Interpretation:** Triapine radiochemotherapy was safe, active, and effective in patients with untreated advanced-stage cervical cancer, worthy of randomized clinical trial study.

## Introduction

Incomplete radiochemotherapeutic treatment responses and poorer cancer-related survivals occur if human papilloma virus (HPV) and abnormal p53 signaling overactivates ribonucleotide reductase in cancers of the uterine cervix (1–8). Human cervical cancer cells overproduce *de novo* deoxyribonucleotides (the building blocks of DNA), overhaul radiochemotherapy-induced DNA damage more easily than other cancer cells, and outlive cytotoxic radiochemotherapy exposures (5–8). Pharmacological blockade of ribonucleotide reductase lowers enzyme catalytic activity, lengthens repair time of radiochemotherapy-induced DNA damage, and elicits cancer cell death (5–8). Enhanced cell lethality after ribonucleotide reductase inhibition may take advantage of a time-critical unmet high *de novo* deoxyribonucleotide demand during low *de novo* deoxyribonucleotide supply (9). In previously conducted clinical trials in untreated patients with advanced-stage cervical cancer, inhibitors of ribonucleotide reductase (e.g., hydroxyurea, 5-fluorouracil, and gemcitabine) have improved radiochemotherapy efficacy (10–12). Another ribonucleotide reductase inhibitor, triapine (3-aminopyridine-2-carboxaldehyde thiosemicarbazone, NSC #663249), has further boosted radiochemotherapeutic activity and efficacy in untreated patients with advanced-stage cervical cancer (1, 2).

Overall, two important anticancer strategies have demonstrated positive clinical outcomes in cervical cancer management: (a) ribonucleotide reductase inhibition (1, 2), and (b) angiogenesis inhibition (13, 14). This study of triapine radiochemotherapy protocols #7336 and #8327 was undertaken to report long-term clinical outcomes and toxicity in selected previously untreated stage IB2–IIIB cervical cancer patients nearly matching the Radiation Therapy Oncology Group protocol #0417 cohort (13).

## Methods

### Patient eligibility

**Figure 1:**
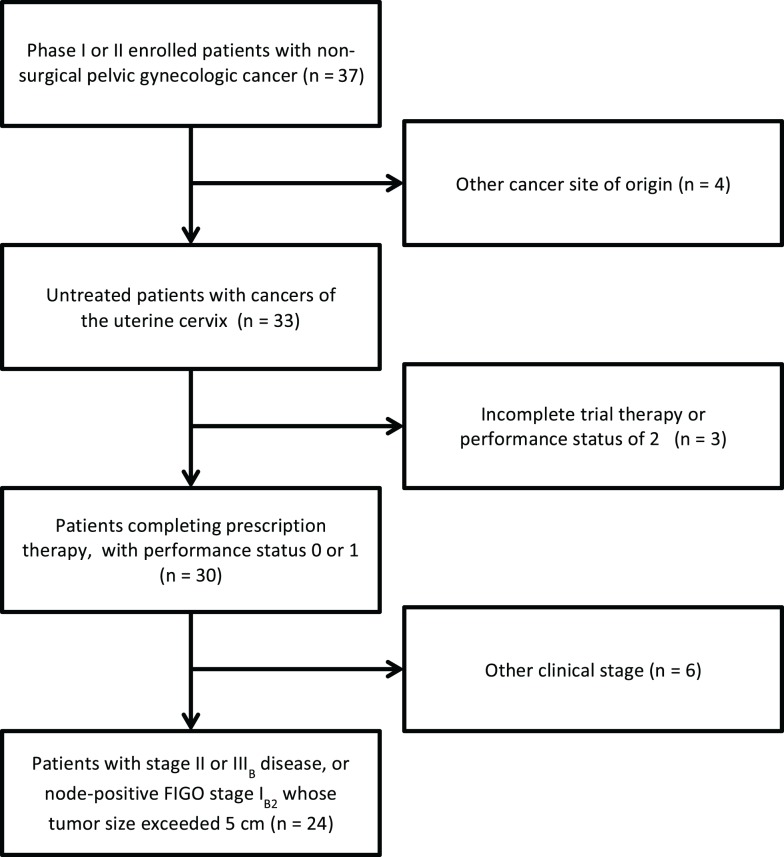
**STROBE diagram for progress through stages of analysis**. STROBE, strengthening the reporting of observational studies in epidemiology.

For the triapine dose-finding phase I protocol #7336, eligible patients had any histologically confirmed primary or recurrent pelvic gynecologic malignancy not amenable to curative surgery. For the triapine efficacy phase II protocol #8327, eligible patients had squamous carcinoma, adenocarcinoma, or adenosquamous carcinoma staged IB2–IVB of the uterine cervix or staged II–IV of the vagina. Prior reports list detailed eligibility criteria (1, 2). Herein, we exclusively report on those enrolled patients with International Federation of Gynecologists and Obstetricians (FIGO) stage II or IIIB disease, or node-positive FIGO stage IB2 whose tumor size exceeded 5 cm (Figure 1; Table 1). All patients provided written informed consent. University Hospitals of Cleveland and Case Western Reserve University (Cleveland, OH, USA) institutional review board approvals were granted for these clinical trials. The Case Comprehensive Cancer Center of University Hospitals of Cleveland and Case Western Reserve University provided oversight for the data and safety monitoring plans.

**Table 1:** **Pretherapy patient and tumor characteristics (*n* = 24)**.

Age (years)
Median	57	
Range	33–68	
Race
Black or African American	9	(38%)
White	15	(62%)
Ethnicity
Hispanic or Latino	2	(8%)
Not Hispanic or Latino	22	(92%)
Performance status
0	23	(96%)
1	1	(4%)
Histology
Squamous	24	(100%)
Adenocarcinoma	0	(0%)
Adenosquamous	0	(0%)
Histologic grade
G1: well differentiated	0	(0%)
G2: moderately differentiated	10	(42%)
G3: poorly differentiated	14	(58%)
Lymph node status
Positive	15	(63%)
Negative	9	(37%)
FIGO stage
IB2	6	(25%)
IIA	6	(25%)
IIB	2	(8%)
IIIA	0	(0%)
IIIB	10	(42%)

*FIGO, international federation of gynecology and obstetrics*.

### Radiation, cisplatin chemotherapy, and triapine treatment

The details of dose-escalation and protocol therapy have been outlined previously (1, 2). Briefly, patients underwent three-times weekly triapine [25 or 50 mg/m^2^ (1 h after radiation on days 1, 3, and 5)] and once-weekly cisplatin [40 mg/m^2^ (prior to radiation on day 2)] chemotherapy during daily four-field pelvic radiation therapy (days 1 through 5). This schedule was repeated for five consecutive weeks (median 45 Gy). An anteroposterior parametrial boost with central pelvic shielding (median 9 Gy) was given during week six, with any missed triapine doses allowed to be made up during this boost. Intracavitary low-dose-rate brachytherapy (median 40 Gy) brought the median point A dose to 85 Gy. No adjuvant therapy was administered in these trials.

### Patient assessments and follow-up

Patients had physical examinations and hematologic, hepatic, and renal function blood testing and baseline computed tomography (CT) or 2-[^18^F]fluoro-2-deoxy-d-glucose positron emission tomography scans (FDG PET/CT) within 28 days before the start of radiochemotherapy. Physical examinations, adverse event assessments (National Cancer Institute Common Terminology Criteria for Adverse Events, version 3.0), and blood work were repeated weekly. Posttherapy physical examinations and adverse event assessments were required at 1 month and at 3 months after completing all radiation therapy. Patients were followed generally every 3 months thereafter by one of the treating physicians. Three-month FDG PET/CT scans were mandatory on the phase II protocol #8327 and optional on the phase I protocol #7336.

### Statistical methods

For this study, a pelvic locoregional relapse was defined as any disease occurring within the pelvic radiation treatment field. Extrapelvic distant disease relapse was defined as any new disease occurring outside of the radiation treatment field, inclusive of para-aortic lymph node relapses. Pathologic confirmation of relapse was not required. The rates of pelvic locoregional relapse were calculated as a proportion and with the 95% confidence interval (CI) determined with no continuity correction. Disease-free survival events were defined as cancer-related death, locoregional relapse, and extrapelvic relapse. Disease-free survival was measured from the start date of radiochemotherapy through the date of first relapse or cancer-related death. Overall survival events were deaths from any cause. Overall survival was measured from radiochemotherapy start date through the date of death. Disease-free and overall survival were calculated by the method of Kaplan and Meier (15).

Toxicity endpoints were protocol-defined and scored according to National Cancer Institute Common Terminology Criteria for Adverse Events, version 3.0 (1, 2). Table 2 identifies protocol-defined adverse events happening during therapy or within the first 30 days posttherapy, and late adverse events occurring greater than 30 days posttherapy.

**Table 2:** **Protocol-defined triapine-attributed treatment adverse events occurring at any time (*n* = 24)**.

	Acute^a^		Late^b^	
	Grade		Grade	
Category	3	4	3	4
Methemoglobinemia	0	0	0	0
Blood/bone marrow	2	0	0	0
Cardiovascular (general)	0	0	0	0
Gastrointestinal	0	0	1	0
Neurology	1	0	0	0
Renal (electrolyte)	1	2	0	0
Genitourinary	0	0	2	0
Worst non-hematologic	2 (8%)	2 (8%)	3 (13%)	0 (0%)
Worst overall	4 (17%)	2 (8%)	3 (13%)	0 (0%)

*^a^Occurring on-treatment or within 30 days of any protocol treatment*.

*^b^Occurring >30 days after any protocol treatment*.

## Results

### Patients

Phase I accrual to protocol #7336 occurred between May, 2006 and August, 2008. Phase II accrual to protocol #8327 transpired between August, 2009, and November, 2011. Thirty-seven patients were recruited to these two protocols from a single institution; 33 patients had squamous, adenocarcinoma, or adenosquamous cancers of the uterine cervix of which 24 comprise this analysis (Figure 1). Of the 13 patients excluded from this analysis, 1 patient had stage IV uterine stromal sarcoma; 3 patients had vaginal cancer; 1 patient did not receive any protocol therapy; 1 patient had a pretherapy Zubrod performance status of 2; 1 patient had clinical stage IIIB but radiographic stage IVB disease and died from an iatrogenic Mallory–Weiss tear prior to brachytherapy; 2 patients had stage IB2 node negative cervical cancer; 1 patient had stage IVA cervical cancer; and 3 patients had clinical stage IVB cervical cancer. Pretherapy demographic and tumor characteristics for the remaining 24 patients are listed in Table 1. Median follow-up time was 3.4 years (range, 0.3–7.6 years). Median follow-up among survivors was 3.8 years (range, 2.2–7.6 years). For the 24 patients reported herein, the median age was 57 years (range, 33–68 years). The majority of the 24 patients had a pretherapy Zubrod performance status of 0 (96%) and either node-positive stage IB–IIA (*n* = 8, 33%) or stage IIIB (*n* = 10, 42%) disease. Although these protocols included non-squamous cervical cancers, all 24 patients under study here had squamous cell cancers of the uterine cervix. Fifteen (63%) had HPV subtype-16 and two (8%) had HPV subtype-18. Seven tumors lacked (*n* = 5) or had unknown (*n* = 2) HPV status by polymerase chain reaction–restriction fragment length polymorphism.

### Adverse events

Adverse events attributable to triapine administration or biologic effect were low in these 24 patients (Table 2). Symptomatic methemoglobinemia [i.e., methemoglobin concentration >20% associated with dyspnea (1, 2)] did not occur in patients in either protocol. Attributable grade 3 or 4 toxicity occurred on-treatment or within 30 days of any protocol therapy in six patients (25%). Attributable late >30-day posttherapy toxicity included two (8%) incidences of grade 3 vaginitis and one (4%) incidence of enterovaginal fistula. No triapine radiochemotherapy treatment administration-related deaths took place.

### Treatment compliance

Compliance with clinical trial protocol-specified radiation and chemotherapy administration was considered satisfactory. Three National Cancer Institute-monitored audits confirmed data timeliness and quality. A total of 342 (95%) of an expected 360 triapine infusions occurred in the 24 patients studied here; there were no triapine infusion-related adverse events.

### Efficacy

**Figure 2:**
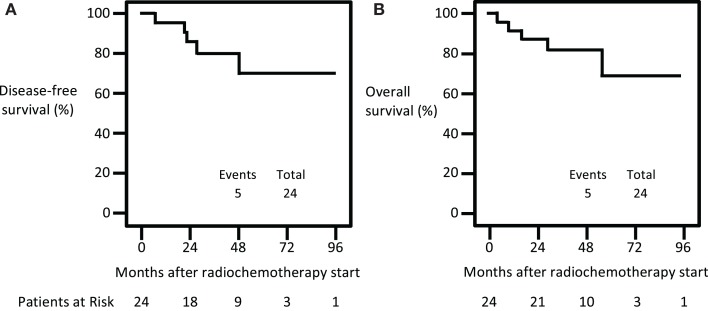
**Kaplan–Meier survival curves for disease-free survival (A) and overall survival (B)**.

The 3-year pelvic locoregional relapse rate, disease-free survival, and overall survival were 4% (95% CI: 0–20%), 80% (95% CI: 71–89%), and 82% (95% CI: 74–90%), respectively. Figure 2 plots disease-free survival and overall survival curves. Nineteen (79%) patients were without treatment failure at the time of last follow-up. Among women in this particular cohort, a single right pelvic sidewall relapse was detected at 8 months after the start of triapine radiochemotherapy. In this patient, a 3-month FDG PET/CT suggested an incomplete pelvic lymph node metabolic response (2). At surgery conducted to palliate non-treatment-related pain, a site of viable extranodal disease was removed surgically from the right pelvic sidewall. Four extrapelvic relapses (i.e., outside the radiation portal) have been recorded: two (8%) para-aortic lymph node relapses; one (4%) abdominal wall relapse; and one (4%) left supraclavicular lymph node relapse. Five (20%) deaths have occurred. Two (8%) of the deaths were attributed to cancer-related factors.

## Discussion

Triapine added to cisplatin radiochemotherapy resulted in a low treatment-related adverse event rate and produced a 3-year disease-free survival rate of 80% (95% CI: 71–89%). These two important clinical trials demonstrate how the supply and demand economics of deoxyribonucleotides affect the degree to which cervical cancers respond to conventional treatment. The majority of cervical cancers have (1) HPVs that hijack ribonucleotide reductase to mint deoxyribonucleotides for replication of viral DNA, or (2) mutations in p53 that abrogate cell cycle restriction checkpoints, free-up ribonucleotide reductase to increase deoxyribonucleotide payout, and replicate DNA unchecked (9). As a consequence of either biologic phenomenon, ribonucleotide reductase subunits M2 and M2b are abundant in cervical cancer cells (3, 4). In this biologic state, ribonucleotide reductase M2 and M2b subunits saturate its catalytic M1 subunit (3, 4), which leads to overproduction of deoxyribonucleotides (5, 6). Because cervical cancers are rich in active ribonucleotide reductase, which can facilitate DNA damage repair (3, 4), patients with (over)active ribonucleotide reductase might have a less than ideal radiochemotherapy treatment response. And yet when ribonucleotide reductase activity is blocked, radiochemotherapy-induced DNA damage persists, cancer cell death occurs, and cervical cancer disease becomes better controlled. Perhaps these concepts are best demonstrated by the 4% rate of pelvic locoregional treatment failure among previously untreated patients enrolled on these two radiochemotherapy trials using triapine to inhibit cervical cancer cell ribonucleotide reductase. Because of these intriguing findings, a National Cancer Institute Cancer Therapy Evaluation Program randomized phase II trial currently tests triapine radiochemotherapy versus cisplatin radiochemotherapy in untreated patients with locally advanced-stage cervical cancer (NCT01835171).

The antitumor consequence of ribonucleotide reductase inhibition has not been the only advancement in cervical cancer treatment. Angiogenesis inhibition has emerged as an effective alternative anticancer treatment strategy for patients with cancers of the uterine cervix and may serve to frame the triapine radiochemotherapy results reported herein. Inactivation of angiogenesis effectors reduces small blood vessel growth, blocks new blood vessel formation, and restores tumor blood nutrient and oxygen supply so that treatments such as chemotherapy and radiation are more deadly to cancer cells (16, 17). Gynecologic Oncology Group protocol #227C studied single-agent bevacizumab (15 mg/kg every 21 days) as an angiogenesis inhibitor in women with recurrent or metastatic cervical cancer and found a 24% rate of disease-free survival at 6 months (18). Phase III testing involved adding bevacizumab (15 mg/kg every 21 days) to cisplatin–paclitaxel or cisplatin–topotecan chemotherapy in patients with metastatic, persistent, or recurrent cervical cancer on Gynecologic Oncology Group protocol #240 (14). Bevacizumab was administered until disease progression or manifestation of unacceptable toxicity. On this trial, a noteworthy 3.7-month improvement in median overall survival (17 vs. 13.3 months) was observed. Also, the Radiation Therapy Oncology Group embarked upon protocol #0417 to evaluate the addition of three bevacizumab doses (10 mg/kg) to weekly cisplatin radiochemotherapy in 49 patients with bulky stage IB2–IIIB cervical cancer (13). No maintenance bevacizumab was administered. The trial showed a 3-year overall survival of 81% (95% CI: 67–90%), disease-free survival of 69% (95% CI: 54–80%), and cumulative incidence of locoregional relapse of 23% (95% CI: 11–35%). The bevacizumab and cisplatin radiochemotherapy grade 3 and grade 4 worst overall toxicity rates were 27 and 10%, respectively.

Because of the exciting clinical outcomes for these two ribonucleotide reductase inhibition clinical trials, a randomized phase II trial of cisplatin radiochemotherapy alone or with co-administered triapine has been put forward for consideration in the National Cancer Institute National Clinical Trials Network program.

### Research in context

#### Systematic review

Our manuscript reports new 3-year safety and efficacy data from clinical trials conducted with triapine radiochemotherapy in untreated patients with advanced-stage IB2–IIIB cancers of the uterine cervix. We searched PubMed with the terms “triapine,” “radiation,” “cervical cancer,” and “clinical trial” for publications between January 1, 1999, and May 1, 2014. Only the original single institution phase I (1) and phase II (2) trials were found – with neither study providing more than 18-month clinical outcome data. We broadened our publication search to include “bevacizumab,” given a publication discussing improved survival with bevacizumab in patients with advanced cervical cancer (14). These three bevacizumab clinical trials were selected to frame the context of our new long-term triapine radiochemotherapy clinical trial data (13, 14, 18).

#### Interpretation

Three-year clinical trial outcomes after three-times weekly triapine added to cisplatin radiochemotherapy in patients with untreated locally advanced-stage cervical cancer demonstrate efficacy results that warrant further clinical trial evaluation. A randomized phase II clinical trial currently recruits untreated patients with stage IB–IVA cancers of the uterine cervix to cisplatin radiochemotherapy with or without triapine (NCT01835171).

## Author Contributions

Charles A. Kunos and Tracy M. Sherertz contributed to the data collection and drafting of this manuscript. This manuscript has been seen, read, and agreed upon in its content by both designated authors.

## Conflict of Interest Statement

The authors declare that the research was conducted in the absence of any commercial or financial relationships that could be construed as a potential conflict of interest.
